# Isolating glomeruli from mice: A practical approach for beginners

**DOI:** 10.3892/etm.2013.1000

**Published:** 2013-03-12

**Authors:** XIAODAN LIU, QIULING FAN, GANG YANG, NAN LIU, DONG CHEN, YI JIANG, LINING WANG

**Affiliations:** Department of Nephrology, The First Affiliated Hospital, China Medical University, Shenyang, Liaoning 110001, P.R. China

**Keywords:** Dynabeads, glomerulus, kidney perfusion, proteomics

## Abstract

A modified procedure for Dynabead perfusion was developed to provide a practical methodology for obtaining large quantities of glomeruli from mice with a high purity. The glomeruli may be useful in exploring the mechanism behind glomerular diseases in conjunction with proteomics. The aim of the study was to save on costs and help researchers, particularly beginners, in the practical application of this method in their studies. Kidneys of C57BL/6 mice were perfused via two different techniques with Dynabeads. The purity and structures of the isolated glomeruli were investigated. The amounts of glomerular protein were measured and the costs of kidney and heart perfusions were compared. There was a 100% success rate at all stages involved in separating the glomeruli of mice via kidney perfusion. The isolated glomeruli remained intact and the purity was 96.7±1.2%. The average amounts of protein in the isolated glomeruli of 8- and 20-week-old mice were 45.6±13.4 and 55.8±17.0 *μ*g, respectively. The cost of glomerular isolation via kidney perfusion was one-fortieth of the cost of isolation via heart perfusion. The described procedure is practical and has a high success rate. The isolated glomeruli of mice were intact and pure and a large quantity was obtained at a lower cost.

## Introduction

Chronic kidney disease is a worldwide public health problem (1,2) and the number of patients with end-stage renal disease is increasing steadily (3). Chronic kidney disease leads to huge health and socio-economic problems. Primary (4,5) and secondary glomerular diseases, including diabetic nephropathy (6,7), are the major causes of end-stage renal disease worldwide. Therefore, the early detection and prevention of glomerular diseases may reduce their global burden. Thus, it is imperative to investigate the pathogenesis of glomerular diseases (8–11).

There are multiple autoimmune strains of mice with glomerulonephritis and mice are also commonly used to create models of diabetes, which develop renal injury similar to human diabetic nephropathy (12). These models are suitable for exploring the mechanisms that lead to kidney disease. However, since the diameters of mouse glomeruli are relatively similar to those of the tubules, it is difficult to isolate pure glomeruli with sieving, as is possible for other animals, including rats (13) and rabbits (14).

Currently, the separation methods available for isolating mouse glomeruli include laser capture microdissection, differential sieving and Dynabead perfusion. Laser capture microdissection has the advantage of high precision; however, the amount of glomeruli obtained is relatively low. Furthermore, with differential sieving it is difficult to isolate mouse glomeruli with high purity. At present, Dynabead perfusion is the only method for separating glomeruli in mice that yields high purity and large amounts; however, the only drawback of this method is that it is expensive (15).

In the present study, glomeruli were isolated from mice via Dynabead perfusion of the kidney and they were of high quality and were isolated at a lower cost. In this study, the course and basic steps are explained, and a practical step-by-step procedure is provided, for isolating mouse glomeruli with Dynabeads. The aim is to aid researchers in the practical application of this methodology in their studies.

## Materials and methods

### Animals

C57BL6 mice, aged 8 weeks, were purchased from the Laboratory Animal Center, China Medical University (Shenyang, China). Mice were housed in plastic cages with free access to food and tap water throughout the experimental period. All mice were maintained in a temperature- and humidity- controlled room (23±3°C; humidity, 50±20%) in the China Medical University, Laboratory Animal Center SPF rodent housing facility with a regular 12 h light/dark cycle, according to the guidelines of the Chinese National Standard (GB 14925-2001). All experiments were approved by a local committee for ethics in animal research.

### Reagents

Collagenase A (product no. 10103578001) was purchased from Roche Diagnostics GmbH (Mannheim, Germany). The 2-D Clean-Up kit and Ettan™ 2-D Quant kit were purchased from GE Healthcare Life Sciences (Piscataway, NJ, USA). Dynabeads M-450 Tosylactivated (diameter 4.5 *μ*m; product no. 140.13) and a magnetic particle concentrator (product no. 123.21D) were purchased from Dynal AS (Oslo, Norway). Cell strainers (100 *μ*m; product no. 352360) were purchased from BD Biosciences (Franklin Lakes, NJ, USA).

### Isolation of glomeruli

**Figure 1:**
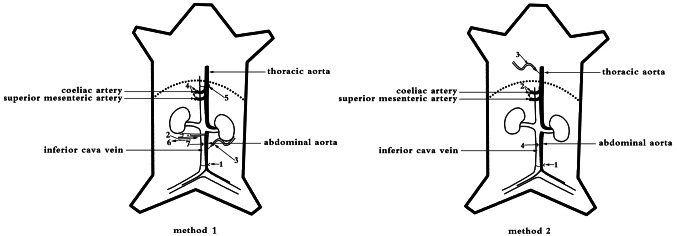
Diagrams of kidney perfusion techniques. Method 1, via the abdominal aorta. Method 2, via the thoracic aorta.

Mice were anesthetized with 10% chloral hydrate (0.03 ml/10 g). The kidneys were perfused via two methods (Fig. 1). Method 1 was performed as follows: i) the distal abdominal aorta and distal inferior cava vein were ligated; ii) the abdominal aorta and inferior cava vein were clipped with vessel clamps below the renal artery and vein; iii) polyethylene tubing (internal diameter, 0.3 mm) was inserted into the middle of the abdominal aorta and fixed in place; iv) the superior mesenteric and coeliac arteries were ligated; v) the proximal abdominal aorta was ligated above the renal artery; vi) the vessel clamp was removed; vii) a hole was cut in the inferior cava vein to ensure venous drainage; and viii) the kidney was perfused with ice-cold sterile phosphate-buffered saline (PBS) through the polyethylene tubing at a constant flow rate of 8.2 ml/min/g kidney to clean the blood vessels of any remaining blood. Method 2 was performed as follows: i) the distal abdominal aorta and distal inferior cava vein were ligated; ii) the superior mesenteric and coeliac arteries were ligated; iii) venous retention needles (24 gauge; BD Biosciences) were inserted into the thoracic aorta and fixed into place; iv) a hole was cut in the inferior cava vein to ensure venous drainage; and v) the kidney was perfused with ice-cold sterile PBS via the venous retention needles at a constant flow rate of 8.2 ml/min/g kidney to clean the blood vessels of any remaining blood.

Following the above mentioned surgical procedures, the kidneys were perfused with Dynabeads. Briefly, Dynabeads were washed prior to use, according to the manufacturer’s instructions. Then, 20 ml Dynabeads at a concentration of 4×10^6^ beads/ml PBS were injected into the kidneys at a constant flow rate of 7.4 ml/min/g kidney. Following perfusion, kidneys were removed, minced into small pieces and digested with collagenase A (1 mg/ml) at 37°C for 30–40 min with gentle agitation. The digested tissue was then gently pressed through a 100 *μ*m cell strainer, followed by intermittent ice-cold sterile PBS flushing. The cell suspension was centrifuged at 200 × g at 4°C for 5 min. The supernatant was discarded and the pellet was dissolved in 2 ml PBS, which was transferred into a 2 ml tube. Glomeruli that contained Dynabeads were isolated by a magnetic particle concentrator and washed at least three times with ice-cold sterile PBS. The entire procedure was performed on ice with the exception of the collagenase digestion. Lastly, the extracted glomeruli were lysed in 2-DE lysis buffer [7 M urea, 2 M thiourea, 4% CHAPS, 2% IPG buffer and 40 mM dithiothreitol (DTT)] and sonicated (30 Hz, 4×5 sec pulses on ice). The lysates were then centrifuged at 12,500 × g at 4°C for 10 min to remove the Dynabeads.

### Measurement of the protein concentration

Protein from glomeruli was purified using the 2-D Clean-Up kit and the protein concentration was determined using the Ettan™ 2-D Quant kit. All samples were stored at −70°C.

### Assessing the isolation of glomeruli

Glomeruli containing Dynabeads were diluted with ice-cold PBS to yield 1 ml and then mixed. Then, 10 *μ*l diluted glomeruli were transferred onto slides with a micropipette and the number of glomeruli and renal tubules were determined by two investigators on an inverted microscope (4× objective lens and 10× ocular lens; Nikon TS100, Tokyo, Japan). The investigators repeated this procedure four separate times and obtained images with a universal microscope (Nikon 80i). Following Dynabead perfusion, the kidneys were fixed with 4% paraformaldehyde, embedded in paraffin, sectioned into 2-*μ*m thick slices, stained with hematoxylin and eosin (H&E) and photographic images were captured under a universal microscope. Renal cortices were rapidly fixed in 2.5% glutaraldehyde, subjected to ferrocyanide-reduced OsO_4_ treatment and dehydrated. Then, plastic infiltration and ultrathin sectioning was performed and the sections were observed and photographed under a transmission electron microscope (EM; JEOL 1200EX). Separated glomeruli containing Dynabeads were fixed in 2.5% glutaraldehyde, osmicated according to the OTOTO protocol (16), dried with hexamethyldisilazane evaporation and photographed under a scanning EM (JEOL T300).

### Statistical analyses

Data are presented as the mean ± standard error of the mean (SEM). P<0.05 was considered to indicate a statistically significant difference. Data were analyzed with SPSS software 15.0 (SPSS Inc., Chicago, IL, USA).

## Results

### Success rate of isolating glomeruli from mice

Kidney perfusions and all steps involved in the isolation of glomeruli in mice were completed with a success rate of 100%.

### Light microscopy

**Figure 2:**
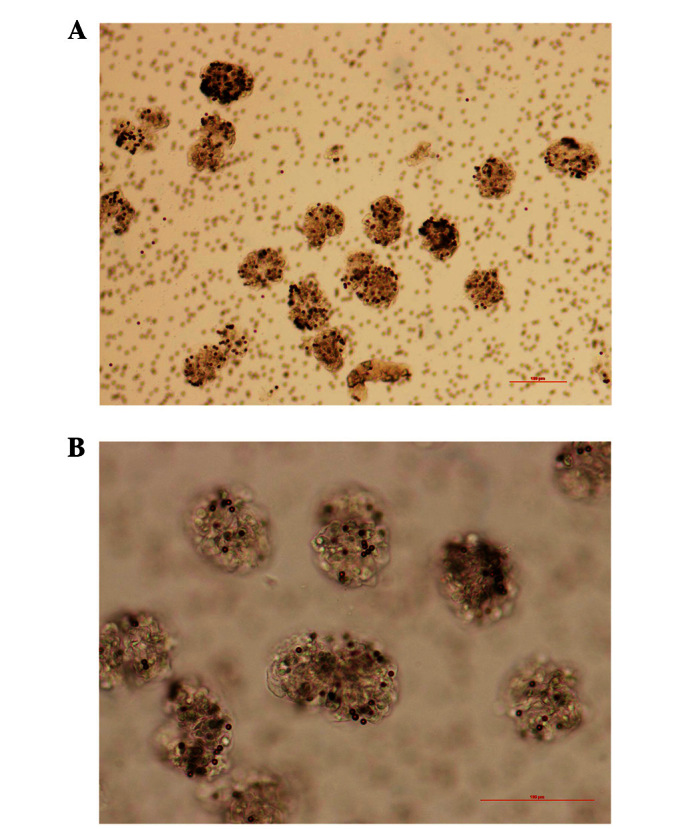
Isolated glomeruli perfused with Dynabeads. Isolated glomeruli occupied the entire visual field. The scale bar represents 100 mm.

Under a light microscope, the isolated glomeruli occupied the entire visual field (Fig. 2). A few glomeruli had part of the afferent and/or efferent arterioles attached and only a few renal tubules were identified with light microscopy. The number of glomeruli was estimated to be 9,960±1,575 at 8 weeks of age and 14,230±2,851 at 20 weeks of age and the purity was estimated to be 96.67±1.16%.

### H&E staining

**Figure 3:**
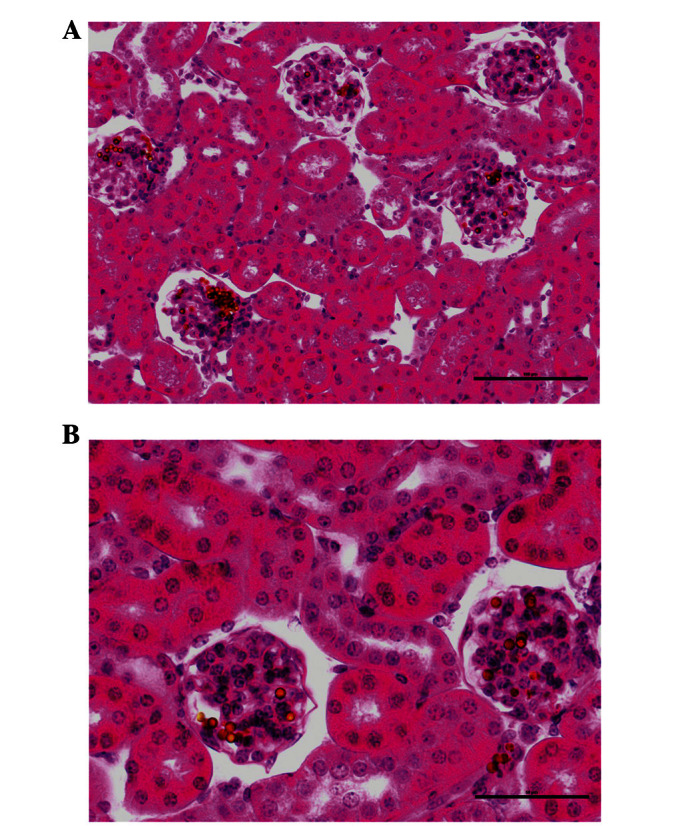
An assessment of Dynabead perfusion with hematoxylin and eosin staining. Dynabeads are located in the majority of the glomeruli. The scale bars represent (A) 100 *μ*m and (B) 50 *μ*m.

Dynabeads were identified in almost all the glomeruli (Fig. 3) and only a few beads were present in the surrounding renal tissues, which were primarily the afferent and/or efferent arterioles.

### Electron microscopy

**Figure 4:**
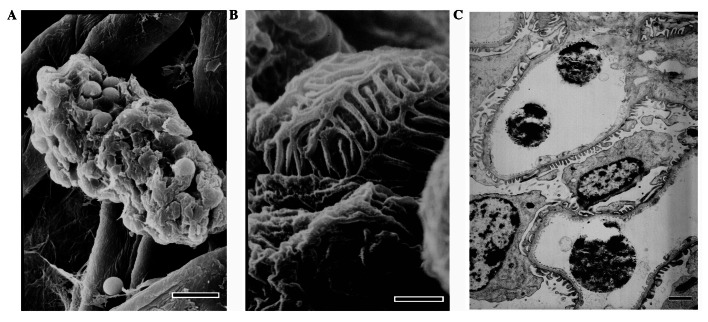
Examination of the quality of isolated glomeruli that contain Dynabeads with electron microscopy. Scanning electron micrographs of (A) an isolated glomerulus that remained intact following the isolation procedure and (B) multiple foot processes that are in a reasonably good shape. (C) Transmission electron micrograph of a transected glomerulus, including the fenestrated endothelial cells, intact foot processes and Dynabeads in capillary lumens. The scale bars represent (A) 10 *μ*m, (B) 1 *μ*m and (C) 2 *μ*m.

Under a scanning EM, closed glomeruli were observed and the structural integrity of the isolated glomeruli was intact. Under a transmission EM, it was identified that Dynabead particles occupied the capillaries, the foot processes of podocytes were in contact with the glomerular basement membrane and the glomeruli structures were intact (Fig. 4).

### Amount of glomerular protein

Twenty mice, 10 mice aged 8 weeks and 10 mice aged 20 weeks, were perfused with Dynabeads. The average amount of protein obtained from the isolated glomeruli of one mouse (from the two kidneys) was 45.6±13.4 *μ*g at 8 weeks of age and 55.8±17.0 *μ*g at 20 weeks of age.

### Effects of different doses of Dynabeads

Ten mice (20 weeks of age) were perfused with either 20 or 30 ml Dynabeads at a concentration of 4×10^6^ beads/ml PBS. There were no significant differences in the average amount of protein obtained from the isolated glomeruli of mice perfused with either 20 or 30 ml Dynabeads (55.8±17.0 vs. 53.7±15.4 *μ*g; P>0.05).

Ten mice (8 weeks of age) were perfused with either 10 or 20 ml Dynabeads at a concentration of 4×10^6^ beads/ml PBS. The average amount of protein obtained from isolated glomeruli from mice perfused with 20 ml Dynabeads was markedly higher compared with that obtained from isolated glomeruli of mice perfused with 10 ml Dynabeads (45.6±13.6 vs. 21.9±6.15 *μ*g; P<0.001).

### Differences in the amount of Dynabeads used following heart and kidney perfusion

The amount of Dynabeads used in the kidney perfusions was one-fortieth of that used in heart perfusions (Table I).

## Discussion

Proteins are the ultimate indicators of biological function. Proteomics has been extensively applied in various fields of medicine, including nephrology (16–20). The application of renal proteomics is likely to aid researchers in gaining an improved understanding of renal pathophysiology and discovering new therapeutic targets. However, the main limitation of glomerular proteomics is obtaining an adequate amount of glomeruli from mice that is also high in purity.

Due to this limitation, current proteomic studies focus on investigating blood and urine proteomics, as well as podocyte and mesangial cell proteomics, which are based on cell culture (21,22). However, the glomerulus is a functional unit with an interconnected organizational structure, coordinated physiological functions and potential interacting pathological changes. Tissues and cells growing in an artificial culture differ greatly from those grown in their original environment and as a result, glomerular proteomics are fundamental. Thus, proteomics at all levels require comprehensive analysis in order to determine the important factors involved and/or correlated with various glomerular diseases. Consequently, it is necessary to develop a practical method for preparing an adequate amount of pure glomeruli, to allow researchers to engage in proteomic research exploring the pathogenesis of glomerular diseases.

In the present study, mouse glomeruli were separated via Dynabead perfusion with a success rate of 100%. The structures of the isolated glomeruli remained intact and the purity was high, while the cost was reduced. The cost of the procedure when the kidneys are perfused, as described in the present study, is one-fortieth of the cost when the heart is perfused, as described by Takemoto *et al*(15). It should be noted that the superior mesenteric and coeliac arteries, which supply blood to the intestines and liver, were ligated to ensure that all the Dynabeads directly entered the kidney. The modification of this step significantly reduced the amount of Dynabeads necessary and consequently reduced the cost. Moreover, the superior mesenteric and coeliac arteries are simple to identify and this modification was easily accomplished. Furthermore, it was identified that even though the number of glomeruli was lower in the 8-week-old mice than in 20-week-old mice, the same amount of Dynabeads was required for perfusion to produce good results.

Kidney perfusion experiments are routinely conducted by researchers studying kidney disease. Since the bore and elasticity of arteries differ and depend on the strain, age or state of the experimental animal, it is difficult for beginners to conduct the surgical procedures involved in such kidney perfusion experiments, particularly when laboratory mice are expensive and a high success rate is required.

Perfusion through the abdominal aorta is common. In the current study, the distal abdominal aorta and inferior cava vein were ligated, and the abdominal aorta and inferior cava vein were temporarily clipped below the renal artery and vein, in order to prevent mice from hemorrhaging during the procedure.

In young mice, the abdominal aorta is thin and it is difficult to insert catheters with a high success rate, particularly for inexperienced researchers. In certain disease models, including KK/Ta mice (23), which are a model of type 2 diabetic nephropathy, the state of the abdominal aorta is poor and the vessel is easily damaged when inserting a catheter. In the present study, the catheter was inserted into the thoracic aorta, which is thicker and easier to handle. Attempts to perfuse the kidneys via the thoracic aorta in mice of different strains and ages were made and a 100% success rate was achieved, even when the perfusion was performed by beginners.

The purity of the isolated glomeruli obtained in the present study was high and consistent with the results of other studies (15,24). The step of isolating glomeruli containing Dynabeads with a magnetic particle concentrator is important for attaining a high purity. Thus, researchers need to ensure that they wash glomeruli at least three times with ice-cold sterile PBS. However, in the present study, the amounts of protein and the numbers of isolated glomeruli were lower than those reported previously (15,24). Subsequent experiments revealed that collagenase A digestion is necessary to detach the glomeruli from their surrounding tissues, otherwise a large amount of glomeruli in undigested tissues would be removed by the cell strainer. Furthermore, in another experiment, the time of collagenase A digestion was prolonged, which increased the amount of protein and the number of glomeruli isolated. Simultaneously, glomerular RNA was also obtained (data not shown).

In conclusion, a useful method for isolating glomeruli from mice in large amounts and with a high purity is presented. The modified procedure is likely to reduce the difficulty in performing the procedure, as well as the cost. Consequently, the modified methodology provides researchers with an opportunity to perform proteomic studies on glomerular diseases.

## Figures and Tables

**Table I:** Amount of Dynabeads used with either heart or kidney perfusion.

Perfusion method	Operational concentration (beads/ml)	Volume/mouse	Number of Dynabead bottles used^b^
Heart^a^	8×10^7^	40 ml	1.6
Kidney	4×10^6^	20 ml	0.04

a Method of Takemoto *et al*(15).

b The original concentration of Dynabeads is 4×10^8^ beads/ml and the volume of Dynabeads is 5 ml per bottle.
